# Active PZT Composite Microfluidic Channel for Bioparticle Manipulation

**DOI:** 10.3390/s19092020

**Published:** 2019-04-29

**Authors:** Tomas Janusas, Kestutis Pilkauskas, Giedrius Janusas, Arvydas Palevicius

**Affiliations:** Faculty of Mechanical Engineering and Design, Kaunas University of Technology, Studentu str. 56, Kaunas LT-51424, Lithuania; tomas.janusas@ktu.edu (T.J.); kestutis.pilkauskas@ktu.lt (K.P.); arvydas.palevicius@ktu.lt (A.P.)

**Keywords:** thermal replication, PZT composite, bioparticle manipulation

## Abstract

The concept of active microchannel for precise manipulation of particles in biomedicine is reported in this paper. A novel vibration-assisted thermal imprint method is proposed for effective formation of a microchannel network in the nanocomposite piezo polymer layer. In this method, bulk acoustic waves of different wavelengths excited in an imprinted microstructure enable it to function in trapping–patterning, valve, or free particle passing modes. Acoustic waves are excited using a special pattern of electrodes formed on its top surface and a single electric ground electrode formed on the bottom surface. To develop the microchannel, we first started with lead zirconate titanate (PZT) nanopowder [Pb (Zr_x_, Ti_1−x_) O_3_] synthesis. The PZT was further mixed with three different binding materials—polyvinyl butyral (PVB), poly(methyl methacrylate) (PMMA), and polystyrene (PS)—in benzyl alcohol to prepare a screen-printing paste. Then, using conventional screen printing techniques, three types of PZT coatings on copper foil substrates were obtained. To improve the voltage characteristics, the coatings were polarized. Their structural and chemical composition was analyzed using scanning electron microscope (SEM), while the mechanical and electrical characteristics were determined using the COMSOL Multiphysics model with experimentally obtained parameters of periodic response of the layered copper foil structure. The hydrophobic properties of the PZT composite were analyzed by measuring the contact angle between the distilled water drop and the three different polymer composites: PZT with PVB, PZT with PMMA, and PZT with PS. Finally, the behavior of the microchannel formed in the nanocomposite piezo polymer was simulated by applying electrical excitation signal on the pattern of electrodes and then analyzed experimentally using holographic interferometry. Wave-shaped vibration forms of the microchannel were obtained, thereby enabling particle manipulation.

## 1. Introduction

Microfluidic systems, which have networks of microchannels that have dimensions ranging from tenths to hundredths of micrometers, are used to manipulate picoliter-level laminar fluid flow, discrete fluid droplets, particles, or cells [1,2,3,4]. Their functional, analytical, and sampling features are attractive for application in biology, life sciences (such as for cytometry), diagnostics lab-on-chip devices, microscale cell culture, etc. [5,6,7,8]. Initially, microchannels of such systems were fabricated in solid substrates (silicon, glass) [9,10,11,12,13], but with the development of novel plastic materials and their fabrication technologies, poly(methyl methacrylate) (PMMA), polystyrene (PS), polycarbonate (PC), and cyclic olefin copolymer (COC) started to be used due to their biocompatibility and more effective fabrication [12,14,15,16,17,18,19].

In this paper, we propose the concept of an active microchannel, i.e., a microchannel formed in piezo polymer, which combines the advantages of plastic materials and the functional ability of the channel to transport droplets due to wave-type deformation of its walls.

Acoustophoresis, a method based on the application of acoustic radiation force for precise manipulation of picoscale fluid droplets or particles, is well known. The application of surface acoustic waves (SAW) is especially promising. Due to the effect of standing acoustic waves (SSAW), particles are patterned across the microchannel, and due to the effect of travelling surface acoustic waves, streaming of the particles or droplets can be obtained [20,21,22,23,24].

Usually, SAW [20,21,22,23] is excited on a LiNbO_3_ substrate by applying AC signal on a pattern of electrodes formed on the surface of the substrate. The authors of [14] reported on the integration of thin lead zirconate titanate (PZT) film with silicon/glass substrate into a microchannel structure and the generation of bulk acoustic wave (BAW) for patterning of the particles across the microchannel.

In the reviewed cases, the microchannel formations were either composed of separate piezo elements (LiNbO_3_ substrate [20,21,22,23] or PZT [24,25]) and nonactive glass, silicon, or polydimethylsiloxane (PDMS) elements. However, none of the works reported on surface microshape or spatial formations from piezo polymer materials being used in microfluidics. Under the effect of electric excitation at certain zones, such formations would enable bidirectional (along and across microchannel) patterning and streaming of particles.

An analysis of existing and future developments of nano/microfluid mechanical systems in [26] clearly shows that the research problem formulated by us is relevant and needs serious research attention. 

**Figure 1:**
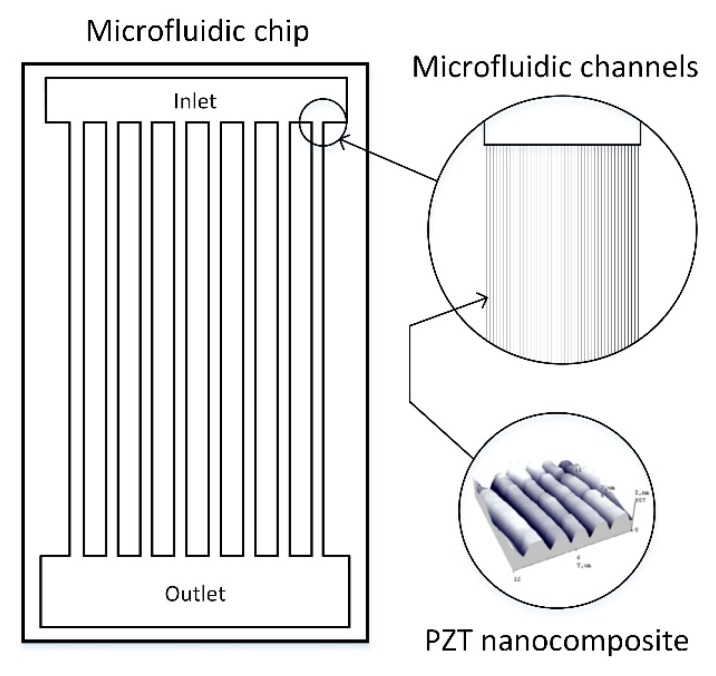
Schematic view of a microfluidic microchip.

In this paper, we present the results of the development of an active microchannel formed in piezo polymer nanocomposite material using the vibration-assisted thermal imprint process. The schematic view of the developed microfluidic chip with a network of microchannels is shown in Figure 1. In the close-up, a fragment of the channels imprinted in PZT nanocomposite is highlighted.

Analyses of its performance in particle trapping, valve, and free pass modes were carried out.

The paper is organized as follows. First, the synthesis of the PZT composite material and fabrication of the PZT composite coating to be used for the microchannel network formation is presented. Then, its composition is analyzed, and its mechanical characteristics are determined. Hydrophobicity analysis of the PZT coatings is carried out by examining their interaction with a water droplet. Next, the vibration-assisted thermal imprint method developed by us is discussed, and the characteristics of the microchannels obtained by it are presented. Finally, a simulation is carried out to examine the behavior of a single microchannel under periodic excitation by COMSOL Multiphysics, and its performance is experimentally tested.

## 2. Materials and Fabrication Process

### 2.1. Synthesis of PZT Composite Material

Lead zirconate titanate nanopowder [Pb (Zr_x_, Ti_1−x_) O_3_] was synthesized by applying the method of oxalate/hydroxide coprecipitation using the following materials: lead(II) acetate Pb(NO_3_)_2_, titanium butoxide Ti(C_4_H_9_O)_4_, zirconium butoxide Zr(OC_4_H_9_)_4_ (80% solution in n-butanol), oxalic acid dihydrate C_2_H_2_O_4_•2H_2_O, 25% ammonia solution, and deionized water. In deionized water (100 ml), 26 g of lead(II) acetate was dissolved. In a separate glass with 500 ml of deionized water, oxalic acid dehydrate (32 g) was dissolved, and the solution was heated up to 50 °C. Then, 5.1 g of titanium butoxide and 7.65 g of 80% zirconium butoxide solution was added drop-wise. The obtained mixture was stirred intensively to obtain a clear yellow solution. This titanium and zirconia alkoxide solution was mixed with the lead acetate solution and alkalized with 25% ammonia solution until pH 9–10 was achieved by continuing to stir the solution for 1 hour. The precipitate of the PZT precursors, i.e., white amorphous, was filtered in vacuum and washed with deionized water and acetone during filtering. Then, the material was dried at 100 °C for 12 h. The obtained powder was calcinated for 9 h at 1000 °C. In the final stage, the PZT powder was milled and mixed with 30% solution of a binding material—PVB, PMMA, or PS—in benzyl alcohol, and a screen-printing paste was obtained. The ratio of the components was defined to be 80% PZT and 20% binder in a dry coating (0.83 g of 30% PVB, PMMA, or PS solution for 1 g of PZT powder). The paste viscosity was adjusted to be 40 ± 5 Pa·s with benzyl alcohol (Brookfield Viscometer, ABZ spindle, 10 rpm, 25 ± 1 °C).

### 2.2. Formation of PZT Composite Material Layer

In the next stage of active microchannel development, the piezo polymer coating in which the active channel would be imprinted was formed on a copper foil. Three coatings with different binding materials were formed and investigated: element 1 with PZT/PMMA coating, element 2 with PZT/PS coating, and element 3 with PZT/PVB coating.

**Figure 2:**
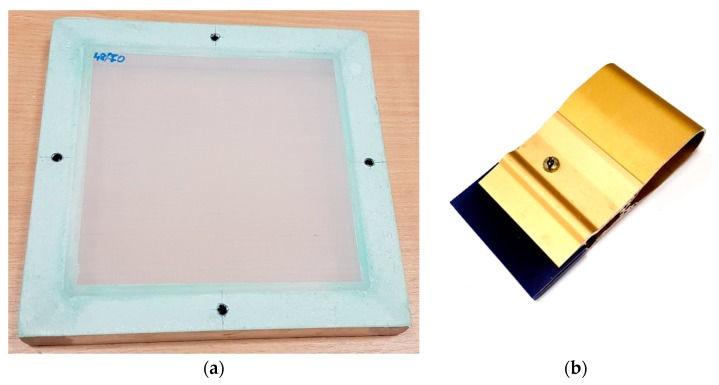
Photo of the polyester monofilament screen (**a**) and layer applicator (**b**).

**Figure 3:**
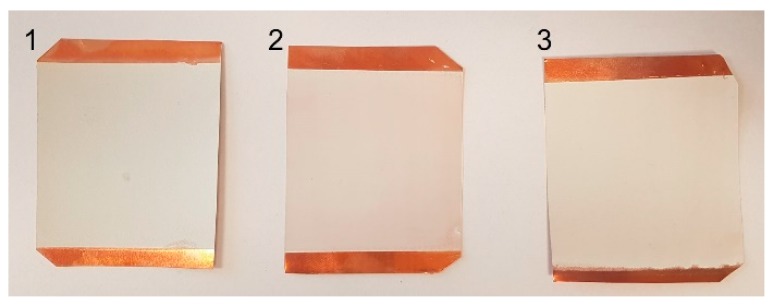
Samples of the coating on copper foil: 1, PZT/poly(methyl methacrylate) (PMMA); 2, PZT/polystyrene (PS); 3, PZT/polyvinyl butyral (PVB).

Using conventional screen printing techniques, the paste was applied to copper foil substrates using a polyester monofilament screen (Figure 2) with 48/70 mesh (Table 1). Then, the coatings were dried at a temperature of 100 °C for 30 min in an electric oven. Images of the coating samples on copper foil are shown in Figure 3. For cases where PZT composite layers of different thickness are needed, parameters of the coatings obtained using other polyester monofilament screens are presented in Table 1. Dimensions of the samples were 55 × 65 mm.

### 2.3. Polarization of the Coating

**Figure 4:**
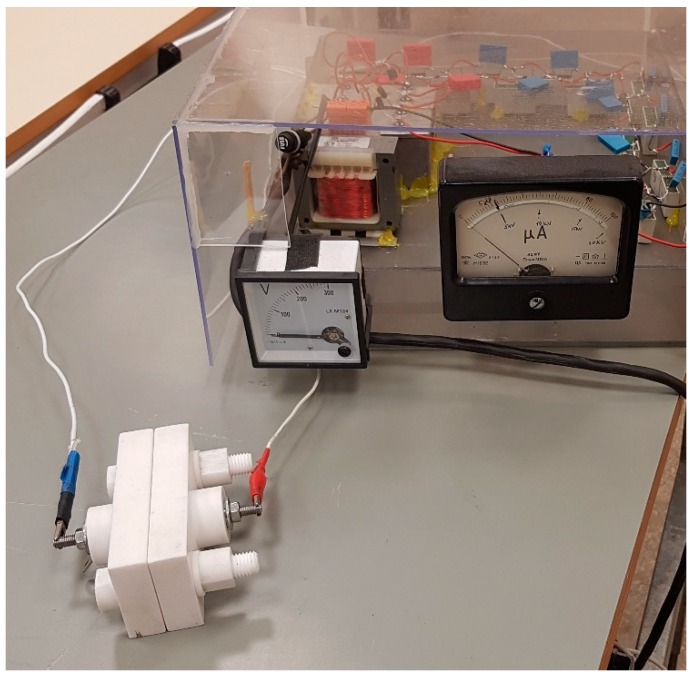
Pole alignment set.

The samples were polarized in order to align the dipole vectors of the PZT grains in it and to obtain the resulting polarization vector perpendicular to the coating plane. Then, a network of active microchannels was formed using a vibration-assisted thermal imprint process. Microchannels, being surface special formations in piezo polymer, undergo deformations under the excitation of electric field due to inverse piezo effect. Polarization of the coating was performed by poling, i.e., electrical pole alignment by applying high voltage on the coating. The copper foil substrate with the coating on it was placed in 5 kV potential for 30 min by fixing it with the help of special brackets in between positive and negative poles (Figure 4). Under the effect of electrical field, PZT grain poles were aligned, resulting in improved voltage characteristics of the piezoelectric coating.

For the analysis of electrical and mechanical properties, 15 × 25 mm size samples were cut from the copper foil substrate with poled PZT polymer coating, and contact wires were attached to them. 

## 3. Experimental Analysis of PZT Coatings

### 3.1. Analysis of Structural and Chemical Composition

Scanning electron microscope (SEM) Quanta 200 FEG integrated with energy-dispersive X-ray spectrometer (EDS) detector X-Flash 4030 from Bruker was used to analyze the structural and chemical composition of the synthesized PZT composite material. In a water steam atmosphere with controlled pressure, three poled elements with different binding materials were examined. Sample 1 was PZT/PMMA, sample 2 was PZT/PS, and sample 3 was PZT/PVB. A 133 eV (at Mn K) energy resolution at 100,000 cps was achieved with a 30 mm^2^ area solid-state drift detector, cooled with Peltier element. Energy differences between quantum states of the system were measured, and the probability of system jumps between these states were determined. X-ray spectroscopy was applied for the analysis of energy distributions.

**Figure 5:**
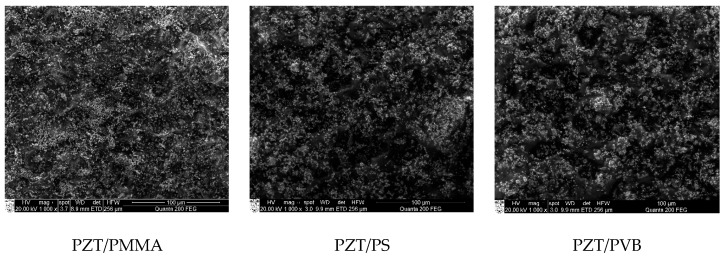
SEM images of the samples with different binders mixed with PZT.

**Figure 6:**
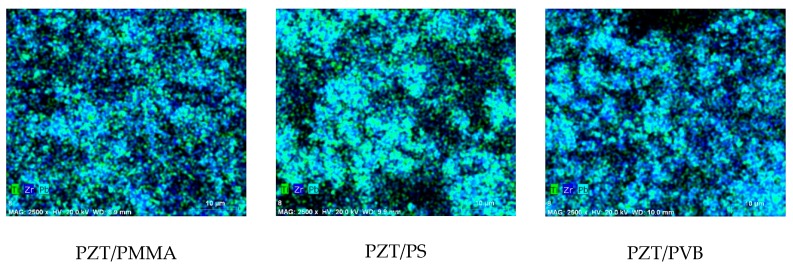
Elemental mapping done with SEM of the samples with different binders mixed with PZT.

The SEM images of the analyzed samples are presented in Figure 5. They reveal the granular structure of sample 1 with grain size of ~1.1 µm in diameter on the surface. The surface of sample 2 was smoother, and its grains were smaller—0.9 µm in diameter. There were 3D structures on sample 3 with empty cavities of 6–8 µm in diameter. The full composition of the elements can be seen in Figure 6. The main composition elements were carbon (C) and zirconium (Zr), which are both good conductors and therefore meet the condition for good piezoelectric properties of the developed novel PZT coatings.

### 3.2. Mechanical and Electrical Characteristics of PZT Coatings

**Figure 7:**
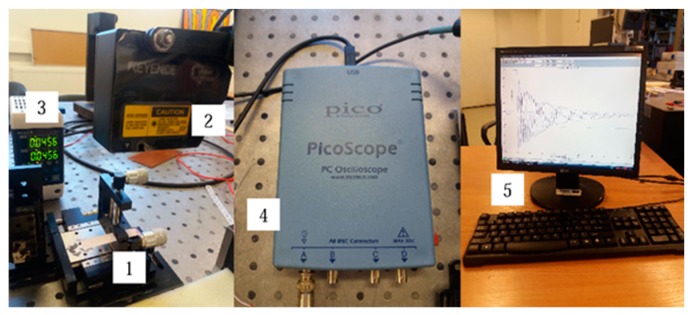
Experimental setup for determination of mechanical and electrical characteristics of the samples: 1, sample holder; 2, LK-G82 sensor head; 3, LK-G3001PV control block; 4, PicoScope oscilloscope; 5, computer.

The mechanical and electrical characteristics of the multilayer foil structure with PZT coating were determined using the COMSOL Multiphysics model and applying the experimentally obtained registered mechanical and electrical parameters of periodic response of the structure. The vibration amplitude was measured with the help of laser triangular displacement sensor LK-G3000, and the generated electrical potential was collected with an USB oscilloscope PicoScope 3424 (Figure 7). By analyzing the first vibration mode by simulation model in COMSOL Multiphysics with the registered data, the mechanical characteristics of every specimen were calculated (see Table 2).

The highest module of elasticity (6.3 GPa) was observed for the PZT nanocomposite with PMMA binding material, while usage of PS and PVB led to lower elasticity modulus of 5.3 and 3.9 GPa, respectively (Table 2). Eighty percent of the nanocomposite material was PZT nanoparticles (Young’s modulus 63 GPa), while the share of binding material was just 20%. Due to this, the modulus of elasticity was reduced approximately 10 times. These results are in agreement with data found in the literature. The Young’s modulus of PMMA is 3.1 GPa [27], the Young’s modulus of PS is 2.7 GPa [28], while PVB has the lowest modulus of elasticity of 50 MPa [29].

**Figure 8:**
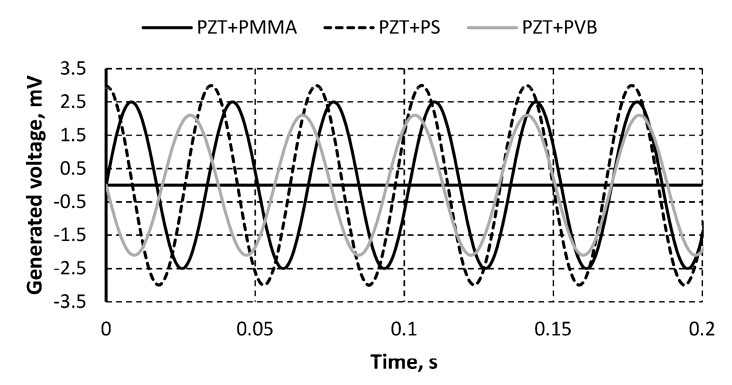
Generated voltage by periodically excited specimens.

Periodic vibrations of the analyzed specimen were excited, and electrical potential due to the piezoelectric effect of the PZT material was generated (Figure 8).

The highest value of generated electrical potential was registered for the specimen with PS as the binding material, with the generated voltage reaching 3 mV. The lowest results was registered for PZT with PVB as the binding material, with a generated voltage of 2.1 mV, while PZT with PMMA as the binding material registered a value of 2.5 mV (Figure 8).

### 3.3. Hydrophobicity Analysis of PZT Coatings

The network of microchannels is a spatial formation imprinted in the developed PZT composite. As it is intended to be used for precise manipulation of droplets, the hydrophobic properties of the surface contacting the manipulated fluid are important. The hydrophobic properties of the PZT composites were analyzed by measuring the contact angle at the interface of the PZT surface and the distilled water droplet.

**Figure 9:**
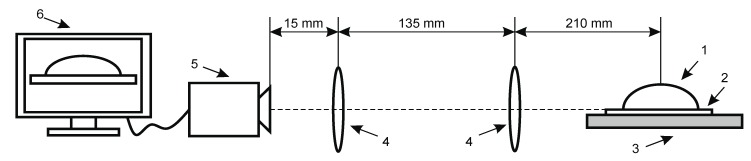
Experimental setup for contact angle measurement of hydrophobic and hydrophilic material: 1, drop on the specimen; 2, analyzed coating; 3, specimen; 4, double convex lenses; 5, Guppy F-503 B&W CMOS camera; 6, computer system for analysis of the captured image.

**Figure 10:**
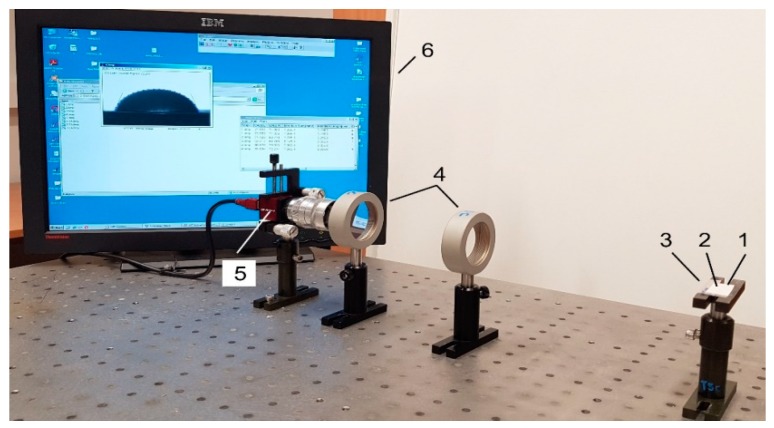
Experimental setup for contact angle measurement: 1, drop on the specimen; 2, analyzed coating; 3, specimen; 4, double convex lenses; 5, Guppy F-503 B&W CMOS camera; 6, computer system for analysis of the captured image.

The structure of the experimental setup for contact angle measurement is shown in Figure 9. All its parts were placed on the surface of a table, which was isolated from external excitation sources. The setup consisted of two optical lenses (focal length of 600 mm) placed between the camera (Guppy F-503 B&W CMOS) and the specimen with a water droplet on its surface. The positioning height of the cameras could be adjusted with respect to the droplet (Figure 10).

Testing was performed in a dark laboratory room with a light setting in which the liquid droplet appeared to be black. This was necessary in order to ensure measurement accuracy and to perform image analysis. 

For contact angle measurement, samples of PZT composites with the three different binding materials were used. Distilled water droplet was used as the liquid.

**Figure 11:**
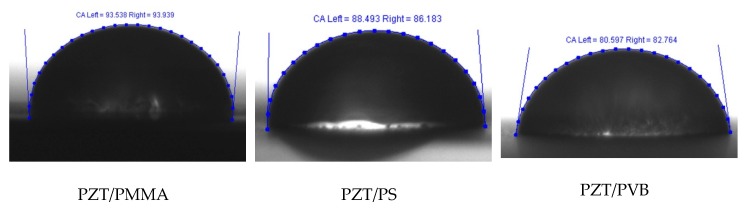
Polynomial fit of the droplet profile through knots starting from the left lower end to the right lower end.

Firstly, for accurate positioning of the droplet image, the height of the specimen holder was adjusted by positioning the specimen parallel to the camera vision field. Then, 0.02 µl droplet of distilled water was diffused from the pipet onto the analyzed surface from a height of 1 cm. For image processing, the open-source software ImageJ provided by Wayne Rasband was used. Drop snake method implemented in its plugin was applied for contact angle measurement. This method is based on using polynomial fit for obtaining the droplet profile curve. Seven knots starting from the left lower end to the right lower end were put along the profile of the droplet, as shown in Figure 11. Then, the droplet profile was approximated as a polynomial curve through these knots, and the contact angle between it and the sample surface was determined.

A water droplet was tested on three different samples of PZT polymer composites with the three different binding materials, i.e., PVB, PMMA, and PS (Figure 10). Each test was done under the same parameters, and the obtained images were analyzed several times.

**Figure 12:**
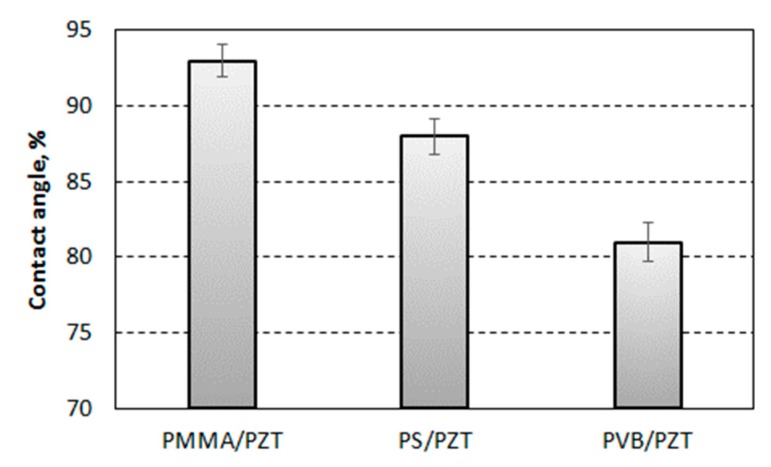
Mean contact angle of a water droplet on PZT composite surface.

Graphical representation of the values of measured contact angle (θ) for different multilayer polymer specimens is shown in Figure 12. The maximum value of the contact angle was observed for PZT + PMMA: 92.94° with a measurement error of ±1°. The lowest contact angle was observed for PZT + PVB: 80.71° with measurement error ±1.3°. The measured value of contact angle for PZT + PS was 88.8° with a measurement error of ±1.16°, which was between the measured values for PMMA and PVB. According to [30,31], polymer materials are considered as hydrophilic if the contact angle of water is less than 90°. Therefore, it could be stated that PMMA was in the range of hydrophobic materials with water, while PVB and PS could be considered as hydrophilic surfaces with water as their contact angle was less than 90°.

## 4. Vibration-Assisted Thermal Imprint of Microchannel Network

The parameters of the network of microchannels formed by the method of vibration-assisted thermal imprint are as follows: length, 20 μm; period, 4 μm; depth, 0.56 μm; width of the land, 2 μm; and width of the ridge, 2 μm.

At present, several microchannel fabrication techniques are known [26], including microdeformation, micromachining, lithography, microelectromechanical systems with deep reactive-ion etching (MEMS (DRIE)), and laser micromachining. Each of them has its own advantages and shortcomings. For example, microdeformation is low cost and quick but is relatively low precision and requires additional processing; micromachining is low cost and has sufficient precision but only allows simplistic shapes to be machined; lithography is a slow process technique; and laser micromachining is a relatively expensive technique, although it can give good accuracy, shape complexity, and process rate parameters.

**Figure 13:**
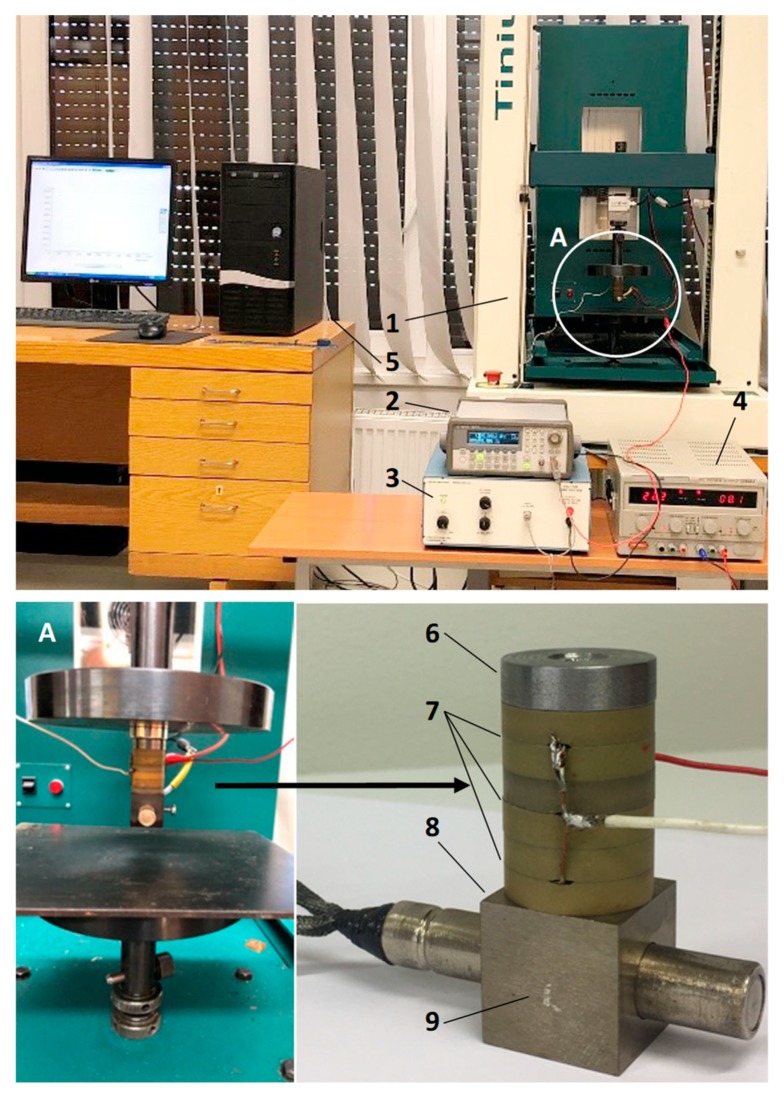
Setup for vibration-assisted thermal imprint of microstructures: 1, Tinius Olsen material testing machine; 2, Agilent 33220A function waveform generator; 3, power source; 4, EPA-104 (Piezo Systems Inc., Woburn, MA, USA) linear amplifier; 5, PC with the installed software for signal processing; 6, steel cap; 7, piezoceramic rings; 8, heating element; 9, cubic-shape front mass.

**Figure 14:**
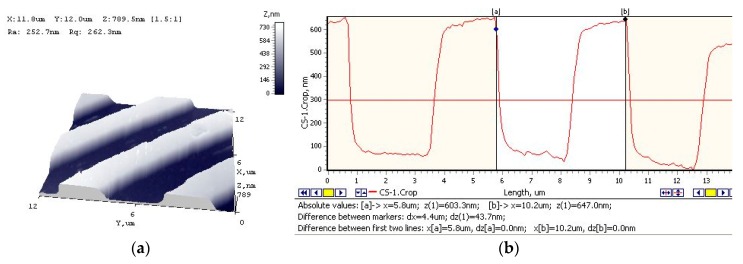
Replica of the microstructure: Atomic force microscope image (**a**) and cross section (**b**) of the replica: profile, lamellar; period, 4 μm; land, 2 μm; ridge, 2 μm; depth, 560 μm.

The proposed method of vibration-assisted thermal imprint combines the advantages of several techniques mentioned above. It is fast like the microdeformation technique but enables much higher precision of the obtained replica and allows more complex geometrical forms to be replicated. A general view of the kit for vibration-assisted thermal imprint of microstructures (or microstructure replication) is shown in Figure 13. It includes a Tinius Olsen material testing machine (1) with a replication unit fixed in it (A), an Agilent 33220A function waveform generator (2), an EPA-104 (Piezo Systems Inc., Woburn, MA, USA) linear amplifier (4) power source (3) of heating element (8), and a PC with the installed software for signal processing (5). The main functional part—the replication unit that is fixed in grip elements of the testing machine—is presented in Figure 13A. Here, the sonotrode above the PZT polymer-coated copper foil can be clearly distinguished. A detailed structure of the sonotrode is also given. To the bottom surface of the cubic-shape front mass (9), a nickel master matrix is attached. High frequency excitation is applied to the front mass with the help of a piezo stack (6-7) attached to its top surface, and it is heated using a heating element (8) perpendicularly traversing it. The dimensions of the cube are 22 × 22 × 22 mm. The piezo stack consists of six piezoceramic (piezoceramics PZT-5H) rings (7) with the following dimensions: outer diameter, 22 mm; inner diameter, 10 mm; thickness, 5 mm and steel cap (6) for creating initial pre-stress of the rings. The imprint process is carried out as follows. The sonotrode with the attached mass matrix is pressed against the surface of the copper foil with PZT polymer coating with the force creating 0.5 bar pressure at the contact under 11 kHz vibrations generated with the help of piezo stack and 1480 temperature achieved using the heating element. After maintaining the pressure for 10 s, the replica of the microstructure is obtained. Geometrical characteristics of its profile are shown in Figure 14.

## 5. Numerical Modeling and Experimental Analysis of Microchannel Performance

**Figure 15:**
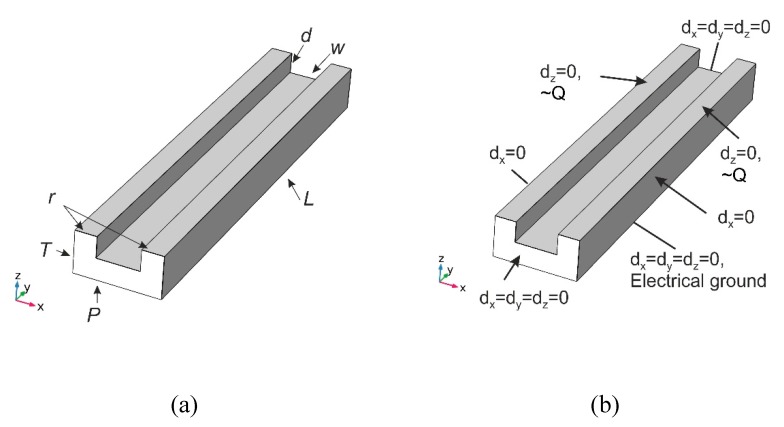
Dimensions (**a**) and boundary conditions (**b**) of the finite element model of microchannel.

The behavior of the imprinted microchannel was investigated numerically using COMSOL Multiphysics 5.2a software. The microfluidic system shown in Figure 1 is periodical. It consists of a number of parallel microchannels with the same geometry (*L* = 20 μm, *P* = 4 μm, *T* = 2 μm, *d* = 0.56 μm, *w* = 2 μm, 2*r* = 2 μm), which connect the outlet and inlet fluid containers. Therefore, just one microchannel (Figure 15) was analyzed in the model with symmetrical boundary conditions (Figure 15b). The left and right surfaces were fixed using rollers; the front and back surfaces were fixed immovably as they were attached to fluid containers. The bottom surface was attached to other components of the sensor; therefore, it was fixed fully and served as an electrical ground. Electrodes were formed on the top of the microchannel system. It was covered by a transparent layer, allowing visual inspection of the fluid flow and ensuring high pressure inside the channel. Vibrations in the network of microchannels were generated by sinusoidal electrical signal of 20 V.

**Figure 16:**
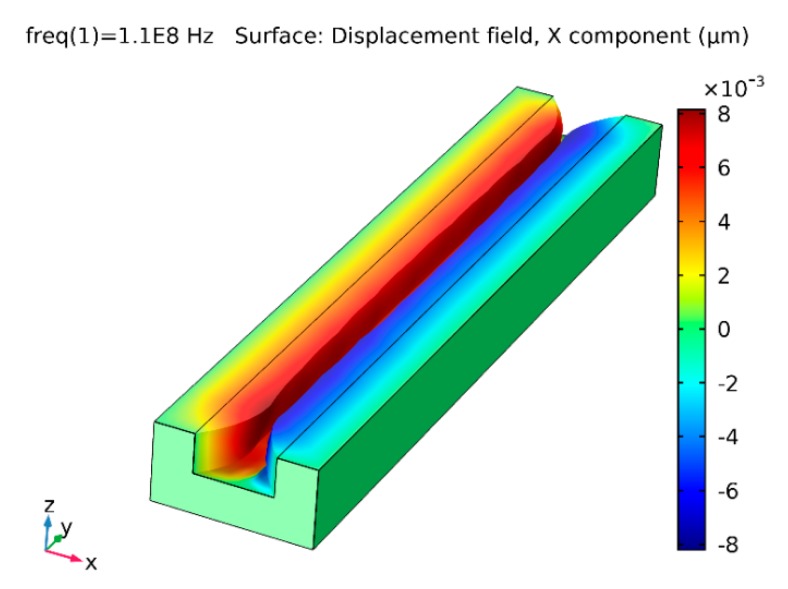
Surface displacement field of microchannel periodically excited at the frequency of 110 MHz: X component visualization.

**Figure 17:**
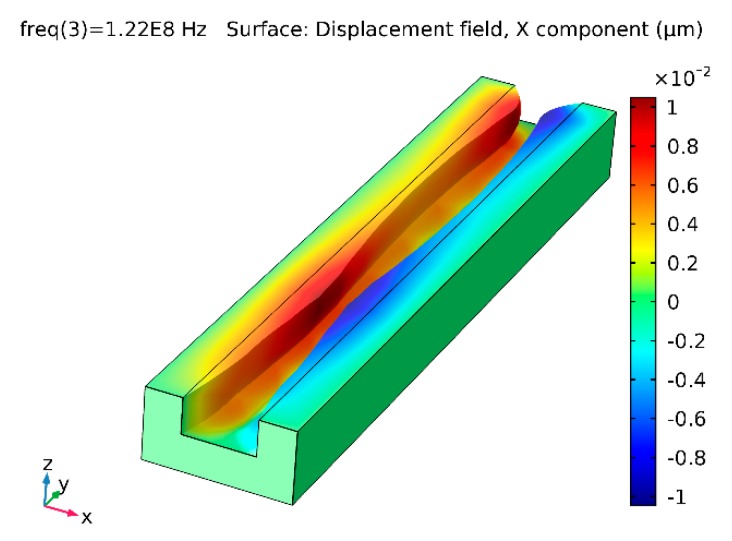
Surface displacement field of microchannel periodically excited at the frequency of 122 MHz: X component visualization.

The results of dynamic response simulation of the microchannel are presented in Figure 16 and Figure 17. Deformation of the system electrically generated at 110 MHz frequency (the first natural frequency) is shown by the X component displacement field (Figure 16). It can be observed that the microchannel oscillated in half-wave mode, i.e., the cross-sectional area increased or reduced in phase through the entire length of the microchannel. Therefore, the microchannel functioned as a mechanical valve, and bioparticles could not pass it as they were stopped near its entrance. The system excited at the frequency of 122 MHz (the second natural frequency of the system) oscillated in full-wave mode, with the two halves of the microchannel along its length oscillating with 180 shift in phase (Figure 17). Thus, the channel was divided into two segments with different concentrations of bioparticles: particles trapped at midpoint (wave node) and particle free at both ends (Figure 17). In the case of no oscillations of the channel, the particles could freely pass it.

**Figure 18:**
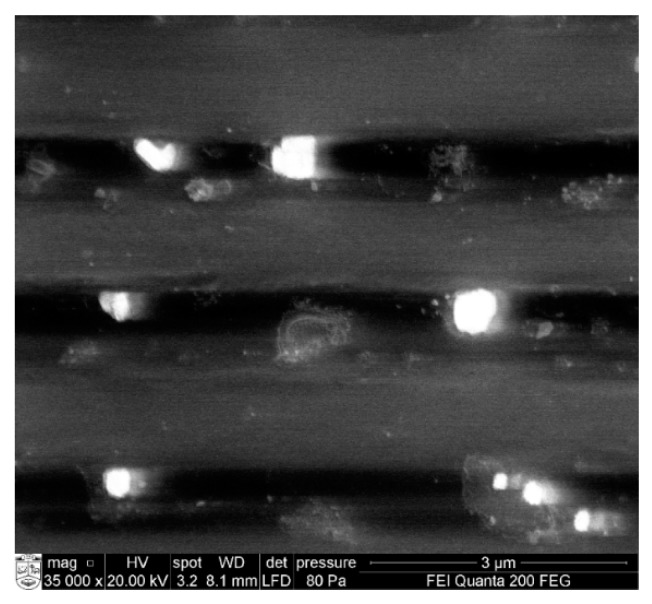
SEM image of bioparticles in motion.

The functioning of the channel in different modes of particle manipulation was analyzed using SEM Quanta 200 FEG, and the SEM images of bioparticle transportation are presented in Figure 18. The first row represents trapping of the particle in midpoint of the channel, the second row represents free pass of the particles, and the third row represents the case of mechanical valve.

Pressure-driven microflow was used for determination of flow characteristics in the microchannel network. The analyzed microchannel network was a periodic microfluidic structure (period, 4 µm; depth, 0.56 µm), which consisted of 100 microchannels. The pressure of 1 bar was maintained in the inlet container. Microflow analysis was performed with three types of liquids: pure water, acetone, and glycerol. In all cases, the calculated Reynolds number was significantly below 1. If the Reynolds number is very small (much less than 1), then the fluid will exhibit Stokes, or creeping flow, where the viscous forces of the fluid dominate the inertial forces. The determined parameters of pressure-driven microflow for different functioning modes of microchannel are presented in Table 3. The experiment with the application of glycerol was not successful as the flow rate obtained was very low, and it was impossible to measure it.

The obtained values of flow rate (Table 3) were in correlation with the vibration shapes of the microchannel. When the channel was vibrating at the first natural frequency of 110 MHz, almost uniform change in its cross-sectional area was observed through the entire length. When the channel was vibrating at the second natural frequency of 122 MHz, the most expressed change in the cross-sectional area was in certain zones of the channel. Thus, the highest flow rate was obtained when no vibrations were generated in the channel as there was no reduction of the cross-sectional area. In case of the first vibration mode, the flow rate was the lowest. This corresponded to the highest change in cross-sectional area. Vibrations at the second natural frequency were the intermediate case, both from the viewpoint of cross-sectional area change and the flow rate. 

The numerical–experimental laser interferometric method was selected and applied for identification of liquid (concentration) flow characteristics in the periodic microstructure. Optical properties of the microfluid were investigated by a nondestructive optical laser diffractometer. He–Ne laser diffractometer (λ = 633 nm) was used in order to register intensities of reflected or transmitted diffraction maxima efficiencies. All liquids were characterized by relative diffraction efficiencies (RDE). The RDE is defined as the ratio of intensity of diffracted light to the *i*-th diffraction maxima (0, ±1, ±2, …) with intensity of the reflected light from the surface without microrelief. The refractive index of the liquids was determined by comparing the theoretical and experimental diffraction efficiencies. Optical properties of the materials used in calculations are presented in Table 4.

**Figure 19:**
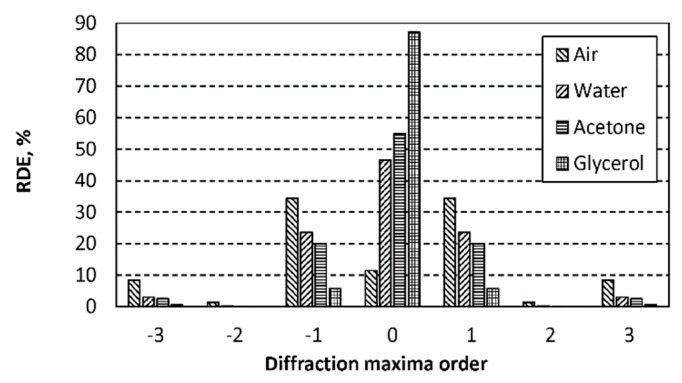
Relative diffraction efficiencies of the reflected light from Al periodic microstructure.

Diffraction efficiencies of the periodic microstructures without an analyte (in air or vacuum) showed that the periodic microstructure (period, 4 µm; depth, 0.56 µm) was designed for operation in reflecting mode, i.e., the first-order diffraction maxima were three times higher than the zero-order diffraction maximum (Figure 19). Therefore, the periodic microstructure in reflecting mode was sensitive enough to refractive index (density) of the analyte. The refractive index changed from 1.33 (pure water) to 1.47 (glycerol), leading to a decrease in the first-order maxima diffraction efficiency by four times, from 24% to 6% (Figure 19). The results validated the idea of identifying the liquid concentration in the periodic microstructures by applying numerical–experimental laser interferometric methods.

## 6. Conclusions

In the present article, the research and development of an active microchannel for improving the effectiveness and accuracy of bioparticle manipulation by acoustic methods are presented.

The main points of the research are as follows:

1. Three different PZT nanocomposite polymers were developed to determine the most suitable option for acoustic field generation. PZT with PMMA as the binding material had an elasticity modulus of 6.3 GPa, PZT with PS as the binding material had an elasticity modulus of 5.3 GPA, and PZT with PVB as the binding material had elasticity modulus of 3.9 GPa.
2. The hydrophobic/hydrophilic properties were experimentally determined by measuring the water contact angle on the surface. The contact angle ranged from 92.94° for PZT + PMMA to 80.71° for PZT + PVB. This indicated that PMMA is a hydrophobic material with water, while PVB and PS showed contact angles of less than 90°, meaning they would be considered as hydrophilic surfaces with water.
3. The proposed vibration-assisted thermal imprint method, which involves thermal replication of microstructures with high-frequency assistance, enabled the formation of a microchannel network with the necessary geometry and precision for bulk acoustic wave generation in the developed PZT nanocomposite.
4. The performance of the microchannel simulated by COMSOL software showed that it could function in three modes: valve mode that was closed for bioparticles when half wavelength oscillations of 110 MHz frequency were generated; bioparticle patterning or trap mode when full wavelength oscillations of 122 MHz frequency were generated; and mode with free pass for bioparticles when no vibrations were generated in the microchannel.
5. The theoretical flow rate values in the microchannels were in agreement with the experimentally obtained flow rate values in the case of pressure-driven microflow when no vibrations were generated. For example, for water, the theoretical value was 0.145 µL/min and the experimentally obtained value was 0.11 µL/min when 1 bar pressure in the outlet container was maintained. The change in the cross-sectional area of the channel when the first mode (110 MHz frequency) and the second mode (122 MHz frequency) vibrations were generated gave the flow rate reduction of up to 0.05 µL/min and 0.08 µL/min, respectively, for water.
6. The developed microchannel system for particle manipulation using bulk acoustic wave in microscale level is in line with the field of single molecule sensor technology and may be used as functional elements in biomedical engineering.

## Figures and Tables

**Table 1:** Properties of polyester monofilament screens and lead zirconate titanate (PZT) composite layer thickness.

Meshed Screen Type	Mesh Opening, µm	Thread, µm	Open Area, %	Mesh Thickness, µm	Theoretical Ink Volume, cm^3^/m^2^	Formed PZT Layer Thickness, µm
32/70	245	70	60.5	108	65	68 ± 1
48/70	130	70	42.3	107	46	60 ± 1
140/34	30	34	22	52	11	25 ± 1

**Table 2:** Mechanical properties.

Binding Material	ρ, kg/m^3^	ζ	f, Hz	ω_n_, rad/s	E, GPa
PVB	6298	0.0052 ± 0.0003	26.5 ± 0.4	166 ± 2.8	3.9 ± 0.3
PMMA	6316	0.0077 ± 0.0006	29.4 ± 0.9	185 ± 5.8	6.3 ± 0.8
PS	6288	0.0080 ± 0.0004	28.3 ± 0.6	178 ± 4.0	5.3 ± 0.4

**Table 3:** Pressure-driven microflow of different modes.

Fluid	Viscosity, cP	Density, g/cm^3^	Theoretical Flow Rate, µL/min	Experimental Flow Rate, µL/min		
				Free Flow	Valve Mode, *f* = 110 MHz	Trapping Mode, *f* = 122M Hz
Pure water	**1**	**1**	0.145	0.11 ± 0.002	0.05 ± 0.001	0.08 ± 0.001
Acetone	0.32	0.79	0.452	0.42 ± 0.004	0.2 ± 0.002	0.31 ± 0.003
Glycerol	1400	1.26	0.0001	-	-	-

**Table 4:** Optical properties of the materials used in calculations.

Materials	Application	Refractive Index	Extinction Coefficient
Air	Superstrate	1	0
Aluminum (Al)	Substrate	1.4495	7.5387
Pure water (H_2_O)	Testing liquid	1.3317	0
Acetone (C_3_H_6_O)	Testing liquid	1.3578	0
Glycerol (C_3_H_5_(OH)_3_)	Testing liquid	1.4707	0

## References

[1] Samandari M., Abrinia K., Sanati-Nezhad A. (2017). Acoustic Manipulation of Bio-Particles at High Frequencies: An Analytical and Simulation Approach. Micromachines.

[2] Zinin P.V., Allen J.S. (2009). Deformation of biological cells in the acoustic field of an oscillating bubble. Phys. Rev. E.

[3] Md Ali M.A., Ostrikov K.K., Khalid F.A., Majlis B.Y., Kayani A.A. (2016). Active bioparticle manipulation in microfluidic systems. RSC Adv..

[4] Hahn P., Leibacher I., Baasch T., Dual J. (2015). Numerical simulation of acoustofluidic manipulation by radiation forces and acoustic streaming for complex particles. Lab Chip..

[5] Pontes B., Ayala Y., Fonseca A.C.C., Romao L.F., Amaral R.F., Salgado L.T., Lima F.R., Farina M., Viana N.B., Moura-Neto V. (2013). Membrane Elastic Properties and Cell Function. PLoS ONE.

[6] Petersson F., Nilsson A., Holm C., Jonsson H., Laurell T. (2004). Separation of lipids from blood utilizing ultrasonic standing waves in microfluidic channels. Analyst.

[7] Augustsson P., Magnusson C., Nordin M., Lilja H., Laurell T. (2012). Microfluidic, label-free enrichment of prostate cancer cells in blood based on acoustophoresis. Anal. Chem..

[8] Hammarstrom B., Laurellab T., Nilssona J. (2012). Seed particle-enabled acoustic trapping of bacteria and nanoparticles in continuous flow systems. Lab Chip.

[9] Ding X., Lin S.C.S., Lapsley M.I., Li S., Guo X., Chan C.Y.K., Chiang I.K., Wang L., McCoy J.P., Huang T.J. (2012). Standing surface acoustic wave (SSAW) based multichannel cell sorting. Lab Chip.

[10] Destgeer G., Ha B.H., Park J., Jung J.H., Alazzam A., Sung H.J. (2015). Travelling surface acoustic waves microfluidics. Phys. Procedia.

[11] Lin S.C.S., Mao X., Huanga T.J. (2012). Surface acoustic wave (SAW) acoustophoresis: Now and beyond. Lab Chip.

[12] Reichert P., Deshmukh D., Lebovitz L., Dual J. (2018). Thin film piezoelectrics for bulk acoustic wave (BAW) acoustophoresis. Lab Chip.

[13] Nava G., Bragheri F., Yang T., Minzioni P., Osellame R., Cristiani I., Berg-Sørensen K. (2015). All-silica microfluidic optical stretcher with acoustophoretic prefocusing. Microfluid. Nanofluid..

[14] McKinstry S.T., Muralt P. (2004). Thin film piezoelectrics for MEMS. J. Electroceram..

[15] Nama N., Barnkob R., Mao Z., Kähler C.J., Costanzo F., Huang T.J. (2015). Numerical study of acoustophoretic motion of particles in a PDMS microchannel driven by surface acoustic waves. Lab Chip.

[16] Baek C., Yun J.H., Wang J.E., Jeong C.K., Lee K.J., Park K.I., Kim D.K. (2016). A flexible energy harvester based on a lead-free and piezoelectric BCTZ nanoparticle–polymer composite. Nanoscale.

[17] Belovickis J., Ivanov M., Samulionis V., Banys J., Solnyshkin A., Gavrilov S.A., Nekludov K.N., Shvartsman V.V., Silibin M.V. (2018). Dielectric, ferroelectric, and piezoelectric investigation of polymer-based P(VDF-TrFE) composites. Phys. Status Solidi (b).

[18] Janusas G., Ponelyte S., Brunius A., Guobiene A., Vilkauskas A., Palevicius A. (2016). Influence of PZT coating thickness and electrical pole alignment on microresonator properties. Sensors.

[19] Janusas G., Ponelyte S., Brunius A., Guobiene A., Prosycevas I., Vilkauskas A., Palevicius A. (2015). Periodical microstructures based on novel piezoelectric material for biomedical applications. Sensors.

[20] Darinskii A.N., Weihnacht M., Schmidt H. (2017). Acoustomicrofluidic application of quasi-shear surface waves. Ultrasonics.

[21] Guo J., Kang Y., Ai Y. (2015). Radiation dominated acoustophoresis driven by surface acoustic waves. J. Colloid Interface Sci..

[22] Sazan H., Piperno S., Layani M., Magdassi S., Shpaisman H. (2019). Directed assembly of nanoparticles into continuous microstructures by standing surface acoustic waves. J. Colloid Interface Sci..

[23] Zheng T., Wang C., Xu C., Hu Q., Wei S. (2018). Patterning microparticles into a two-dimensional pattern using onecolumn standing surface acoustic waves. Sens. Actuators A.

[24] Zhang A.L., Zha Y. (2014). The breakup of digital microfluids on a piezoelectric substrate using surface acoustic waves. IEEE Trans. Ultrason. Ferroelectr. Freq. Control.

[25] Xu D., Cai F., Chen M., Li F., Wang C., Meng L., Xu D., Wang W., Wu J., Zhen H. (2019). Acoustic manipulation of particles in a cylindrical cavity: Theoretical and experimental study on the effects of boundary conditions. Ultrasonics.

[26] Kumar A.U., Javed A., Dubey S.K. (2018). Material selection for microchannel heatsink: Conjugate heat transfer simulation. IOP Conf. Ser. Mater. Sci. Eng..

[27] Xua J., Lia Y., Geb D., Liua B., Zhua M. (2011). Experimental investigation on constitutive behaviour of PVB under impact loading. Int. J. Impact Eng..

[28] Lerch B.A., Thesken J.C., Bunnell C.T. Polymethylmethacrylate (PMMA) Material Test Results for the Capillary Flow Experiments (CFE). https://ntrs.nasa.gov/search.jsp?R=20070030192.

[29] Pianigiani M., Kirchner R., Sovernigo E., Pozzato A., Tormen M., Schift H. (2006). Effect of nanoimprint on the elastic modulus of PMMA: Comparison between standard and ultrafast thermal NIL. Microelectron. Eng..

[30] Ma Y., Cao X., Feng X., Ma Y., Zou H. (2007). Fabrication of super-hydrophobic film from PMMA with intrinsic water contact angle below 90. Polymer.

[31] Kumar P., Khan N., Kumar D. (2016). Polyvinil butyral (PVB), versatile template for designing nanocomposite/composite materials: A review. Green Chem. Technol. Lett..

